# Radiation lays down covering fire for viral shock troops

**DOI:** 10.18632/oncotarget.1187

**Published:** 2013-07-19

**Authors:** Joan N. Kyula, Aadil A. Khan, Michelle J. Wilkinson, David Mansfield, Kevin J. Harrington

**Affiliations:** Department of cancer biology, Institute of Cancer Research, London; Department of cancer biology, Institute of Cancer Research, London; Department of cancer biology, Institute of Cancer Research, London; Department of cancer biology, Institute of Cancer Research, London; Department of cancer biology, Institute of Cancer Research, London

In a recent study of combined vaccinia virus (VV) and radiation therapy (RT) in BRAF-mutant melanomas, we dissected the precise events by which RT enhances viral cytotoxicity. Our studies revealed that the complex interplay between RT, virus and cell signaling pathways caused a completely novel synthetically lethal form of tumor cell death. We initially sought to assess the effect of combining a VV (GLV-1h68) and RT in melanoma cell lines with different genetic backgrounds (BRAF-mutant, RAS-mutant and BRAF/RAS-wild-type). We hypothesised that RT would induce stress responses in all tumor genotypes and activate host survival pathways to create an environment favorable to viral replication and subsequent oncolysis. Contrary to our expectations, enhanced tumor cell kill with the combination of virus and RT only occurred in BRAF-mutant melanoma cells. In mechanistic studies, it was clear that VV triggered activation of the c-Jun N-terminal Kinase (JNK) pathway and tumor necrosis factor-α (TNF-α) signaling in all genotypes. In BRAF-mutant cells, virus-induced TNF-α production was associated with anti-apoptotic/pro-survival effects, as previously described with MEK inhibitor-induced cytotoxicity [1]. Remarkably, when radiation was combined with VV, JNK and TNF-α signaling were shut down and this triggered significant apoptotic death – but only in cells with a BRAF mutation. We were able to mimic this ability of RT to enhance virally-mediated cytotoxicity by switching off JNK by small molecule inhibition (SP600125) or siRNA-mediated gene silencing, but again this was only possible in BRAF-mutant cells. This represents an unprecedented synthetically lethal interaction between radiation and VV that is explicable in terms of manipulation of cell survival pathways. Remarkably, the enhanced cell death seen in BRAF-mutant melanoma was associated with significantly reduced viral production, suggesting that “oncolysis” is a complex process that cannot be explained purely in terms of viral replication. It is hoped that similar studies with vaccinia (or other oncolytic viruses) in particular tumor-specific genetic contexts may reveal other clinically translatable synthetically lethal combinations. For now, future studies of VV and radiation (for local disease) or small molecule inhibitors (for systemic disease) should be considered. GLV-1h68 and its clinical equivalent ‘GLONC-1’ are Lister strain oncolytic VV that have been attenuated by disruption of viral thymidine kinase and hemagglutinin genes and altered with galactosidase, glucuronidase and Renilla luciferase/green fluorescent protein gene insertions. GLONC-1 is currently in Phase I single-agent clinical trials using intravenous, intrapleural or intraperitoneal delivery. Single-agent VV is known to interact with the mitogen-activated protein kinase [2] and Akt pathways [3] to foster a cellular environment conducive to viral replication. The antitumor activity of GLV-1h68 in combination with other anti-cancer modalities has been profiled extensively in preclinical studies. This is important because a number of recent preclinical and translational clinical studies have shown that oncolytic virotherapy may be particularly effective in augmenting the anti-tumor activities of chemotherapy and radiation therapy [4-7]. Most studies published to date have revealed that combining virotherapy and standard anti-cancer agents leads to increased viral replication and enhanced apoptotic/autophagic cell death, although the underlying mechanisms have often been obscure. Combinations with traditional therapeutics are becoming more relevant as means of optimizing patient benefit as oncolytic viruses continue to move closer to regulatory approval. Indeed, it was this growing confidence in the potential benefits of combination therapies that led us to perform these studies. Two recent clinical reports have further energised the field of oncolytic virotherapy. In the first, a granulocyte-macrophage colony-stimulating factor (GM-CSF)-expressing Wyeth strain VV (pexastimogene devacirepvec, Pexa-Vec) demonstrated single agent activity in a randomized phase II trial in patients with hepatocellular carcinoma (HCC). Anti-tumor activity was mediated by both direct oncolytic and indirect immunological mechanisms and survival was related to the dose of administered virus [8]. As a direct result, Pexa-Vec was granted orphan drug status for HCC by the US Food and Drug Administration in 2013 (http://online.wsj.com/article/PR-CO-20130508-915494.html). The second study, with a GM-CSF-expressing herpes simplex virus type 1 (talimogene laherperepvec, T-Vec), was presented at the recent American Society of Clinical Oncology meeting. Four hundred thirty-six patients were randomized (2:1) to single-agent T-Vec or subcutaneous GM-CSF, resulting in a significantly greater durable response rate and median time to treatment failure for the virotherapy arm (http://chicago2013.asco.org/immunotherapies-offer-bright-prospects-advanced-melanoma). Again, both oncolytic and loco-regional/systemic immunotherapeutic activity was documented. In light of these studies, it is important to recognize that Pexa-Vec and T-Vec merely represent two regiments of a larger army of targeted biological agents that includes reovirus (pelareorep, Reolysin^™^), various adenoviruses, coxsackie virus and other herpes and VV vectors. In that vein, VV appear to be particularly promising candidate anti-cancer agents. As we have shown, elucidation of intracellular mechanisms may uncover other avenues of attack, through which combination therapies can be exploited for greater anti-tumour efficacy. Further therapeutic alliances might be sought by genetic manipulation of the viral genome itself to deliver a therapeutic payload and form a ‘coalition of killing’. The potential of targeted, multi-modality therapy centred on the biology of viral oncolytics is an emerging, yet exciting, field of investigation and highlights the crucial role of understanding viral biology. In the words of Sun Tzu (“The Art of War”): “*We cannot enter into alliances with neighbouring princes until we are acquainted with their designs*.”
